# Global trends and current status in pheochromocytoma: a bibliometric analysis of publications in the last 20 years

**DOI:** 10.3389/fendo.2023.1167796

**Published:** 2023-08-23

**Authors:** Bi-ling Huang, Qi Liu, Yuan-yuan Teng, Shu-qin Peng, Ze Liu, Ming-liu Li, Jie-yu Liang, Yi Zhang, Min Wang

**Affiliations:** ^1^ Department of Endocrinology, Xiangya Hospital, Central South University, Changsha, Hunan, China; ^2^ National Clinical Research Center for Geriatric Disorders, Xiangya Hospital, Central South University, Changsha, Hunan, China; ^3^ Department of Orthopaedics, Xiangya Hospital, Central South University, Changsha, Hunan, China

**Keywords:** pheochromocytoma, neuroendocrine tumour, bibliometric analysis, global trends, current status, Web of Science

## Abstract

**Objective:**

Pheochromocytoma is a rare catecholamine-producing neuroendocrine tumour originating from the chromaffin cells of the adrenal medulla or extra-adrenal paraganglia. However, there are few bibliometric studies on Pheochromocytoma. Therefore, this study was employed to summarize the global trends and current status in pheochromocytoma by bibliometric analysis.

**Materials and methods:**

The Web of Science (WOS) core collection database was searched for publications relating to pheochromocytoma from 2001 to 2021. Bibliometric analysis was used to examine the data, and Microsoft Excel was utilized to create bar graphs. In addition, VOSviewer was used to carry out co-authorship analysis, co-citation analysis and co-occurrence analysis. CiteSpace was used to analyze the keywords citation bursts.

**Results:**

A total of 8,653 publications published in 1,806 journals by 38,590 authors in 6,117 organizations from 100 countries/regions were included in our study. Among them, USA was the leading countries in terms of total publications and sum of time cited, whereas Eunice Kennedy Shriver Natl Inst Child Hlth & Hum was the leading institutions. The main publications for pheochromocytoma-related articles were *Journal of clinical endocrinology &metabolism*. Pacak karel and Eisenhofer Graeme were the main contributing authors. The studies on pheochromocytoma could be grouped into five clusters: Treatment, Mechanism, Etiology, Radiology and Hormones study. Moreover, the radiology study, etiology study and some specific keywords such germlines mutation, mesenchymal stem-cells, autophagy, neuroinflammation, neurotoxicity, and hemodynamic instability, may become the hot spots of future.

**Conclusion:**

Although the number of articles on pheochromocytoma has fluctuated slightly over the past 20 years, there has been an overall upward trend. In general, precision medicine research on pheochromocytoma, especially metastatic pheochromocytoma, in terms of diagnosis, treatment, and etiology will be a hot research topic in the future. This study helps to understand the research perspectives, hot spots and trends of pheochromocytoma and provide new insight and a basis for future pheochromocytoma research quickly.

## 1 Introduction

Pheochromocytoma is a rare catecholamine-producing neuroendocrine tumour originating from the chromaffin cells of the adrenal medulla or extra-adrenal paraganglia (1). The diagnosed incidence of pheochromocytoma is 2-8 per million (2, 3). According to the latest 2017 World Health Organization (WHO) classification of endocrine tumours, all pheochromocytomas have metastatic potential, which has replaced the previous term “malignant” (4). The patients with pheochromocytoma often present with paroxysmal symptoms of elevated catecholamine production: headache, palpitations, diaphoresis, facial pallor, anxiety, tremors, and hypertension (5).Compare with other endocrine cancers, pheochromocytoma has the highest heritability or genetically known causes. Around 30-40% of patients with pheochromocytoma were suffered from germline mutations, whereas up to 50% of them have somatic mutations in the same genes (3, 6). Therefore, genetic testing is advised as routine check for pheochromocytoma. Currently, surgery is the first therapy options for non-metastatic pheochromocytoma, whereas few established therapy choices were existed for metastatic pheochromocytoma. Therefore, the novel therapeutic approaches are desperately needed (7). In recent years, radiotherapy, classical chemotherapy and different targeted therapy were increasingly applied (6). The lifelong follow-up and establish individualize therapy are needed for the patients with a pheochromocytoma history and asymptomatic mutation carriers. Since pheochromocytoma was firstly described by Edwin Beer in 1937, great progress has been made regarding the mechanisms, diagnostics, and therapies of pheochromocytoma. Therefore, a comprehensive report with an intuitive overview and trends in the pheochromocytoma-related research is quite needed.

Bibliometric analysis is a novel scientific tool for assessing the global trends and current status, and evaluating contributions in a particular research field by countries, journals, organizations and authors (8, 9). Additionally, the information visualization and bibliometric analysis can forecast the hotspots and trends within a certain research field, which can provide reliable and useful references for future researchers getting new insight and deepening the field (8, 10). However, there was no bibliometric analysis conducted in the area of pheochromocytoma-related research yet, and the prediction of research hotspots has never been discussed. Therefore, this study aims to assess and summarize the global trends and current status in the pheochromocytoma research field. By analyzing pheochromocytoma-related publications in the past 20 years, we hope this study can help researchers identify present status and future trends quickly.

## 2 Materials and methods

### 2.1 Data Sources

The Web of Science (WOS) Core Collection database was used for the search, which included the Science Citation Index Expanded (SCI-Expanded) and Social Sciences Citation Index (SSCI).

### 2.2 Search Strategy

**Figure 1:**
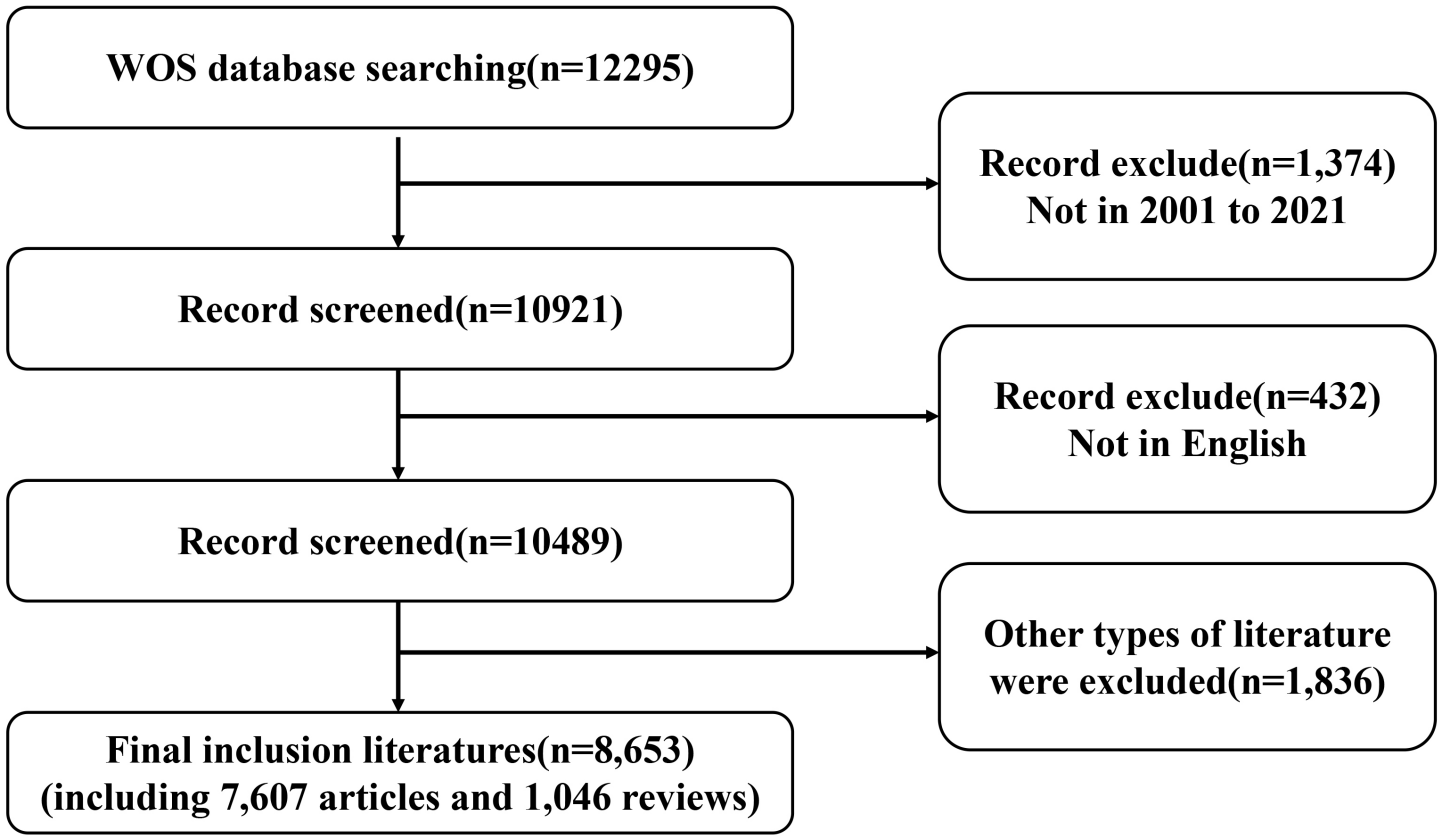
The flowchart of data filtration processing and excluding publications.

All the literature were retrieved in WOS on September 26, 2022. The search terms were TS = (“Pheochromocytoma” OR “Pheochromocytoma, Extra-Adrenal”). The documents type was limited to “Article” and “Review”, with language restriction to English. The time span of the publications was restricted to the years from 2001 to 2021. As shown in **Figure 1**, a total of 8653 papers were ultimately included.

### 2.3 Data collection

Full records and cited references, including titles, publication year, nationalities, authors, institutions of authors, journals of publication, funding sources, abstracts, keywords, total number of publications, sum of citations and average number of citations per item were extracted from the retrieved literature. Data based on bibliometric characteristics were downloaded from WOS and imported into VOSviewer (v.1.6.18), CiteSpace (6.1.R3) and Microsoft Excel 2019 for analysis.

### 2.4 Bibliometric Analysis

VOSviewer (v.1.6.18), CiteSpace (6.1.R3), and Microsoft Excel 2019 were used to conduct the bibliometric analysis and visualization (11, 12). The number of publications (Np) and the cited frequency (Cf) are indicators for bibliographic analysis (13). Np and Cf are two essential indexes for evaluating the efficacy of research; Np is used to assess production capacity, and Cf measures impact. What’s more, the scientific contribution of a researcher can be assessed using the H index. It denotes that a researcher has produced H articles, and each of those papers has received at least H citations from other publications (14). Additionally, it can also define the publication output of a country/region, institution or journal (15).

VOSviewer was used to carry out co-authorship, co-citation, and co-occurrence analysis and to create network visualization maps. co-authorship analysis is a method to assess the connectivity of items based on the quantity of co-authored publications. When two items are both cited in another article, the co-citation analysis approach is used to determine how closely two items are related based on how often they are cited together. Items with a greater total link strength show that the journals/publications have a larger global influence. A co-occurrence network visualization is created by counting the number of publications whose titles or abstracts contained the same group of keywords. Nodes are used in the network visualization to represent the items. The size of the node is affected by the amount of publications related to the item. The estimated distance and thickness between two nodes reveal that how closely linked the items are. The cluster to which an item belongs determines the color of the item.

CiteSpace was used to analyze dual maps of journals, keyword timeline, reference timeline, keywords bursts and reference bursts, to help with the visual assessment of knowledge field and developing trends. Additionally, can highlight some particular crucial documents in the area’s evolution as well as the overall state of a study field.

All the study’s bar charts were statistically analyzed and graphed using Microsoft Excel 2019. Additionally, the H-index and impact factor (IF) were retrieved from the Web of Science in October 2022.

## 3 Results

### 3.1 Global trends of publications in pheochromocytoma

**Figure 2:**
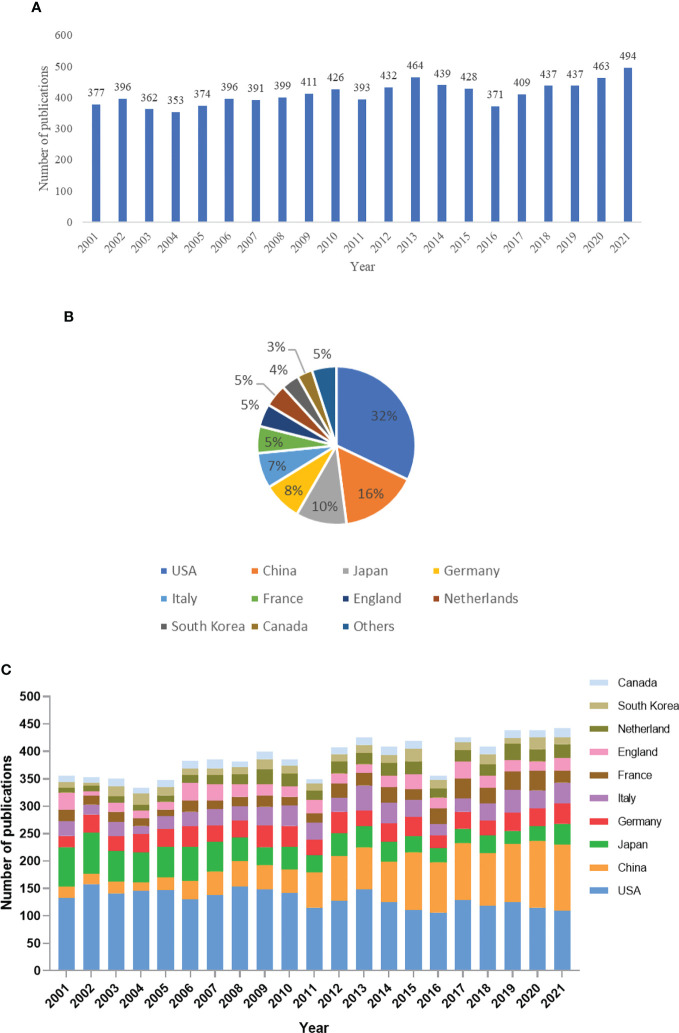
Global trends of publications on Pheochromocytoma in the last 20 years. **(A)** Number of publications per year. **(B)** The total number and percentage of publications from the top ten countries/regions. **(C)** Pheochromocytoma-related publications of the top ten countries/regions over time.

A total of 8,653 pheochromocytoma-related literature, including 7,607 articles and 1,046 reviews published from 2001 to 2021, were included in our study. The number of publications on pheochromocytoma has been relatively stable over the last 20 years (**Figure 2A**). The total number and percentage of publications from the top ten countries were shown in **Figure 2B**. Among them, USA produced the most literature (32%), followed by China (16%), Japan (10%), Germany (8%), Italy (7%), France (5%), England (5%), Netherlands (5%), South Korea (4%) and Canada (3%). The USA and China account for about half of the total number of articles published. **Figure 2C** shows the changes in the number of articles published by each country over time. The number of publications of China increase gradually, while the number of Japanese publications declined steadily. Moreover, the number of publications published by other countries remain stable.

### 3.2 Analysis of the contribution of countries/regions

**Figure 3:**
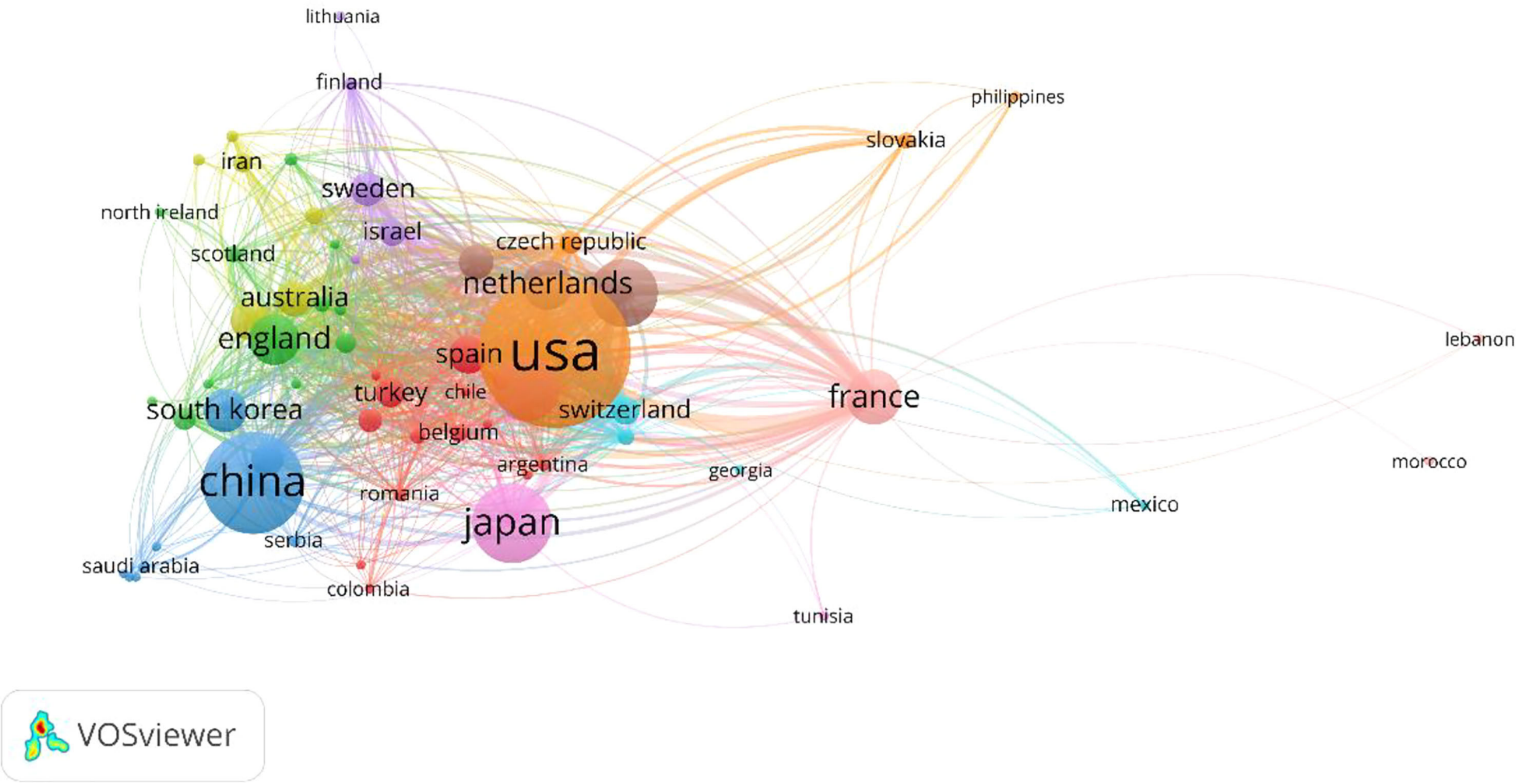
Cooperation among different countries in Pheochromocytoma in the last 20 years. Countries are colour-labelled, and those with strong interactions were grouped together into the same colour cluster.

The top 10 countries in terms of the number of publications were presented in **Table 1**. USA published the most among countries with 2,777 documents and 39.24 average citations, followed by China, Japan, Germany, Italy, France, England, Netherlands, South Korea and Canada. In addition, Netherlands and France have the highest average number of citations at 53.70 and 52.25, respectively. What’s more, the H-index of the USA (571) is also the highest among these countries, followed by England (379) and Italy (301). Moreover, **Figure 3** displays the relationship between 63 selected countries (the minimum number of documents for a country is over five). USA (total link strength = 1,657), Germany (total link strength = 1,040), Netherlands (total link strength = 784), France (total link strength = 762), Italy (total link strength = 745), England (total link strength = 563), Spain (total link strength = 553), Poland (total link strength = 424), Switzerland (total link strength = 379) and Australia (total link strength = 350) were the top 10 countries by total link strength.

**Table 1:** Top 10 countries in the Pheochromocytoma research field.

Rank	Country	Publications	Citations	Average Citation	H-index
1	USA	2,777	108,969	39.24	571
2	China	1,371	24,835	18.11	168
3	Japan	899	22,227	24.72	238
4	Germany	682	28,810	42.24	300
5	Italy	627	23,089	36.82	301
6	France	477	24,924	52.25	278
7	England	402	19,140	47.61	379
8	Netherlands	401	21,536	53.70	275
9	South Korea	314	7,692	24.50	157
10	Canada	271	9,775	36.07	300

### 3.3 Analysis of the contribution of institutions

**Figure 4:**
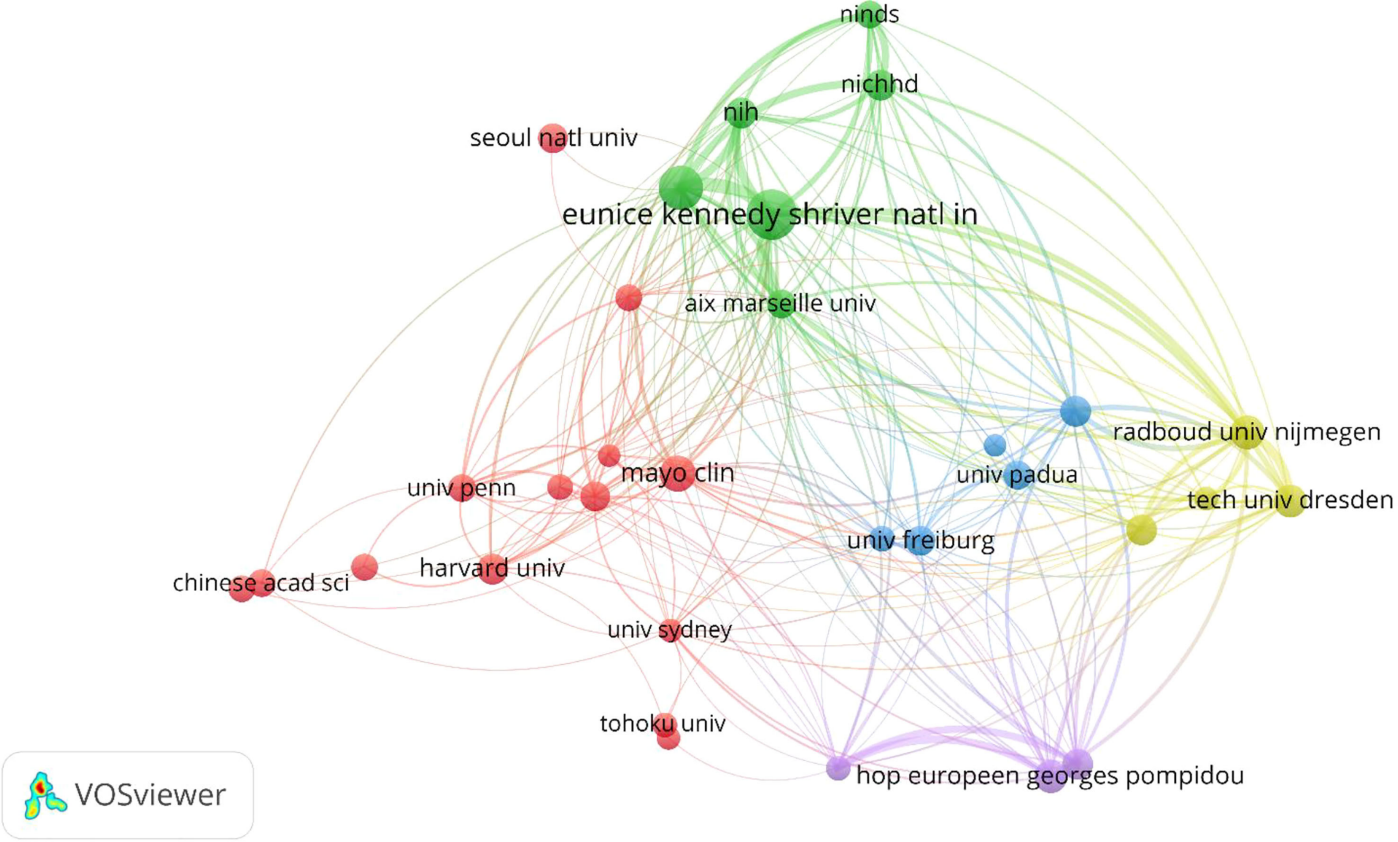
Co-authorship analysis Network visualization of the 32 identified institutions in Pheochromocytoma, with the minimum number of publications was set to 50.

The relationship between the 820 institutions (the minimum number of documents for a country is over fifty) is shown in **Figure 4**. Following are the top 10 institutions by total link strength: Eunice Kennedy Shriver Natl Inst Child Hlth & Hum (total link strength =680), National Cancer Institute (total link strength = 548), Radboud University Nijmegen (total link strength = 532), Hospital Europeen Georges Pompidou (total link strength = 503), Dresden University of Technology (total link strength=462), University of Paris 5 (total link strength = 401), Florence University (total link strength = 389), University of Wurzburg (total link strength=305), National Institutes of Health (total link strength = 297) and Aix-Marseille University (total link strength=295). In light of co-authorship analyses, Eunice Kennedy Shriver Natl Inst Child Hlth & Hum demonstrated greater cooperation than other institutions. As shown in **Table 2**, Eunice Kennedy Shriver Natl Inst Child Hlth & Hum published the most among institutions, with 207 documents and 42.19 average citations, followed by National Cancer Institute, Mayo Clinic, Radboud University Nijmegen, Hôpital Européen Georges Pompidou, Dresden University of Technology, Harvard University, National Institute of Child Health and Human Development, Florence University and National Institutes of Health. In addition, the National Institute of Child Health and Human Development and Florence University have the highest average number of citations at 83.48 and 81.84, respectively. Of these institutions, the first, second, third, seventh, eighth and 10th productive institutions in the Pheochromocytoma research field are all from the United States. While the fourth, fifth, sixth and ninth productive institutions in the field of pheochromocytoma research are from the Netherlands, France, Germany and Italy, respectively.

**Table 2:** Top 10 most productive institutions in the Pheochromocytoma research field.

Rank	Institute	Country	Publications	Citations	Average Citation
1	Eunice Kennedy Shriver Natl Inst Child Hlth & Hum	USA	207	8,734	42.19
2	National Cancer Institute	USA	166	8,217	49.50
3	Mayo Clinic	USA	116	6,919	59.65
4	Radboud University Nijmegen	Netherlands	103	5,347	51.91
5	Hôpital Européen Georges Pompidou	France	98	7,501	76.54
6	Dresden University of Technology	Germany	98	2,341	23.89
7	Harvard University	USA	90	5,421	60.23
8	National Institute of Child Health and Human Development	USA	89	7,430	83.48
9	Florence University	Italy	88	7,202	81.84
10	National Institutes of Health	USA	88	4,270	48.52

### 3.4 Analysis of journals

The top 10 journals in terms of the number of publications were presented in **Table 3**. *Journal of clinical endocrinology &metabolism* published the most among journals with 218 documents, followed by *Journal of neurochemistry*, *Endocrine-related cancer*, *Journal of biological chemistry*, *PoS One*, *Clinical endocrinology*, *Hormone and metabolic research*, *European journal of endocrinology*, *Endocrine pathology* and *Neuroscience letters*. What’s more, the *Journal of clinical endocrinology &metabolism* publish the most documents with the highest average citations of 72.70. Thus, *Journal of clinical endocrinology &metabolism* is the leading journal at Pheochromocytoma worldwide, corresponding to the highest total number of publications and average citations.

**Table 3:** Top 10 journals in the Pheochromocytoma research field.

Rank	Source	Publications	Citations	Average Citation
1	Journal of clinical endocrinology &metabolism	218	15,849	72.70
2	Journal of neurochemistry	113	4,035	35.71
3	Endocrine-related cancer	110	3,869	35.17
4	Journal of biological chemistry	96	5,536	57.67
5	Plos one	85	2,353	27.68
6	Clinical endocrinology	82	2,316	28.24
7	Hormone and metabolic research	74	1,613	21.80
8	European journal of endocrinology	73	3,498	47.67
9	Endocrine pathology	72	1,393	19.35
10	Neuroscience letters	63	1,406	22.32

**Figure 5:**
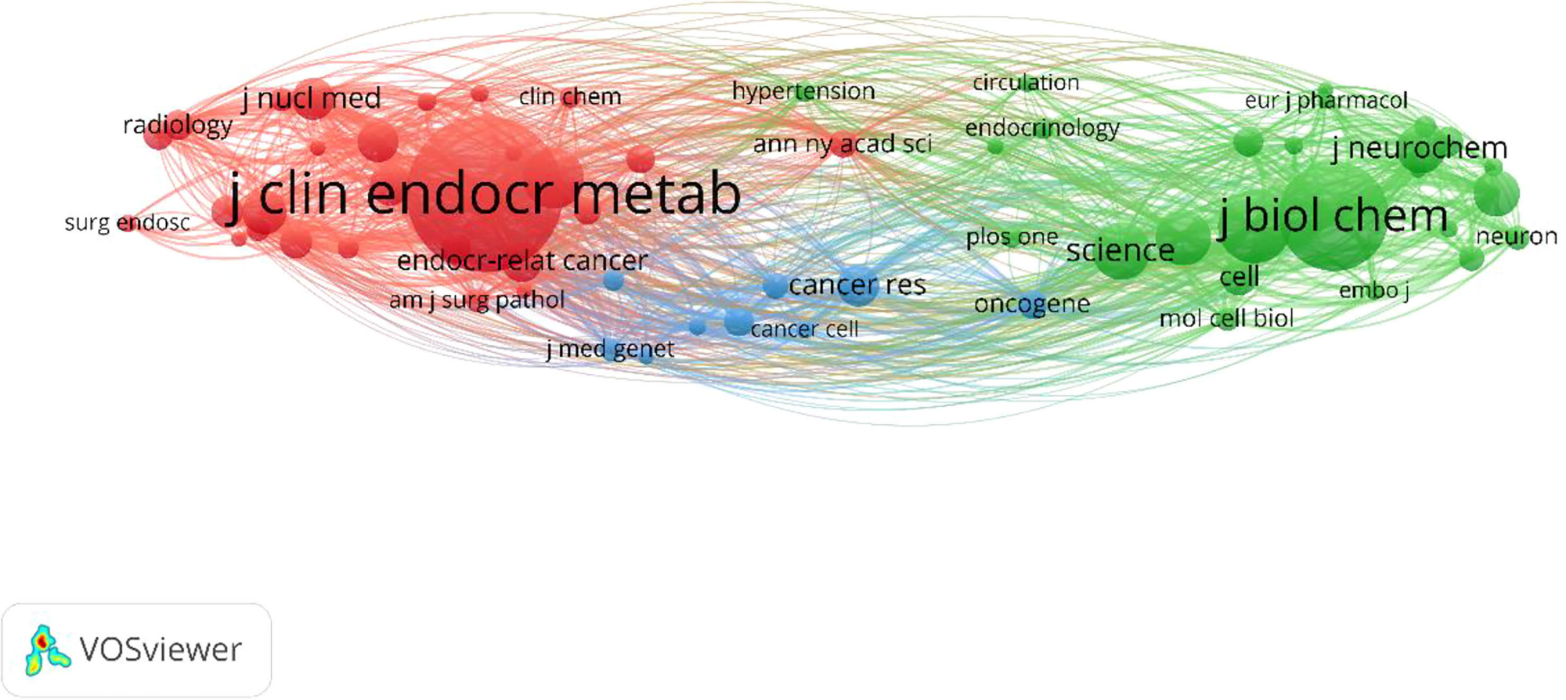
Co-citation analysis and network visualization of the 61 identified journals in Pheochromocytoma, with the minimum number of citations of journals was set to 1,000.

**Figure 5** shows the relationship between the 1,000 recognized journals in terms of overall link strength (a journal must have at least 1,000 citations). Following are the top 10 journals by total link strength: *Journal of clinical endocrinology &metabolism* (792,658 total link strength), *Journal of biological chemistry* (475,863 total link strength), *Proceeding Of The National Academy Of Sciences of The United States Of America* (325,254 total link strength), *New England Journal Of Medicine* (264,347 total link strength), *Science* (258,436 total link strength), *Nature* (242,439 total link strength), *Cancer Research* (198,716), *Journal of neurochemistry* (195,718 total link strength), *Clinical endocrinology* (189,798 total link strength) and *Endocrine-related cancer* (188,615 total link strength). Co-citation analysis revealed that the *Journal of clinical endocrinology &metabolism* was the most popular journal in pheochromocytoma-related research worldwide.

**Figure 6:**
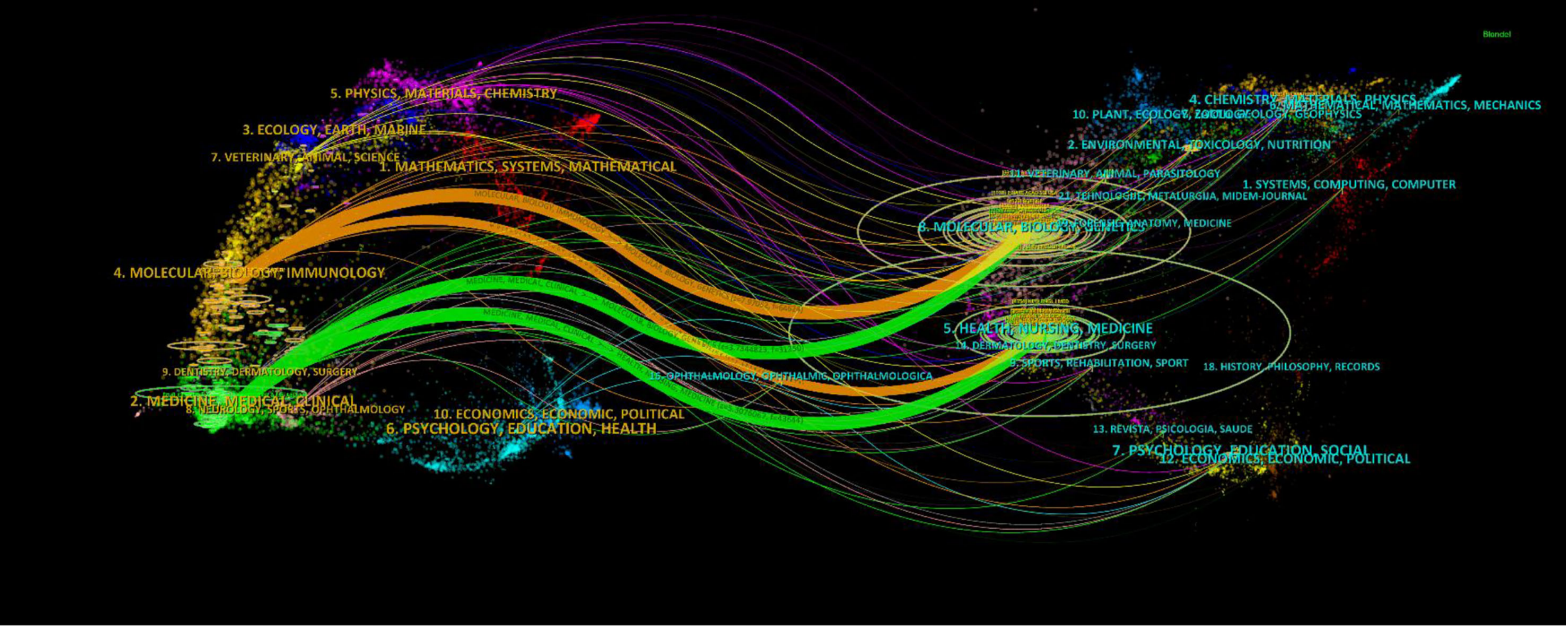
The dual-map overlay of journals on Pheochromocytoma. The citing journals are on the left, the cited journals are on the right, and the colored path represents the citation relationship.

Additionally, the distribution of subjects in scientific journals is shown by the dual-map overlay of journals (**Figure 6**). The locations of the citing and cited journals were on the left and right, with colored paths designating citation links. The four main citation routes for pheochromocytoma were displayed in **Figure 6**. The journals in molecular biology, immunology, and genetics had the strongest citation correlations.

### 3.5 Analysis of authors

**Table 4** listed the top 10 authors according to the quantity of publications. Pacak karel took first place with 301 publications, followed by Eisenhofer Graeme (115 publications), Gimenez-roqueplo Anne-paule (67 publications), Taieb David (60 publications), Lenders Jacques w.m. (52 publications), Timmers Henri j.l.m (48 publications), Robledo Mercedes (41 publications), Jimenez Camilo (41 publications), Mannelli Massimo (40 publications) and Neumann Hartmut p. h. (40 publications). In terms of average citation, Gimenez-roqueplo and anne-paule have the highest average citation rate. As for the H-index, Pacak karelalso ranks first (104), follow by Eisenhofer, Graeme (84) and Gimenez-roqueplo, anne-paule (80). Among these highly productive authors, the first and eighth most productive are from the United States; The second and tenth most productive authors are from Germany; The third and fourth authors of high productivity are from France; The fourth and fifth authors of high yield were from the Netherlands; The authors of the seventh and ninth highest productivity are from Spain and Italy, respectively.

**Table 4:** Most important authors in the Pheochromocytoma research field.

Rank	Author	Country	Publications	Citations	Average Citation	H-index
1	Pacak, karel	USA	301	17,344	57.62	104
2	Eisenhofer, graeme	Germany	115	6,199	53.90	84
3	Gimenez-roqueplo, anne-paule	France	67	5,608	83.70	80
4	Taieb, david	France	60	1,990	33.17	43
5	Lenders, jacques w.m.	Netherlands	52	3,454	66.42	63
6	Timmers, henri j.l.m	Netherlands	48	2,241	46.69	64
7	Robledo, mercedes	Spain	41	1,713	41.78	50
8	Jimenez,camilo	USA	41	1,495	36.46	40
9	Mannelli, massimo	Italy	40	2,248	56.20	73
10	Neumann, hartmut p. h.	Germany	40	1,613	40.33	73

**Figure 7:**
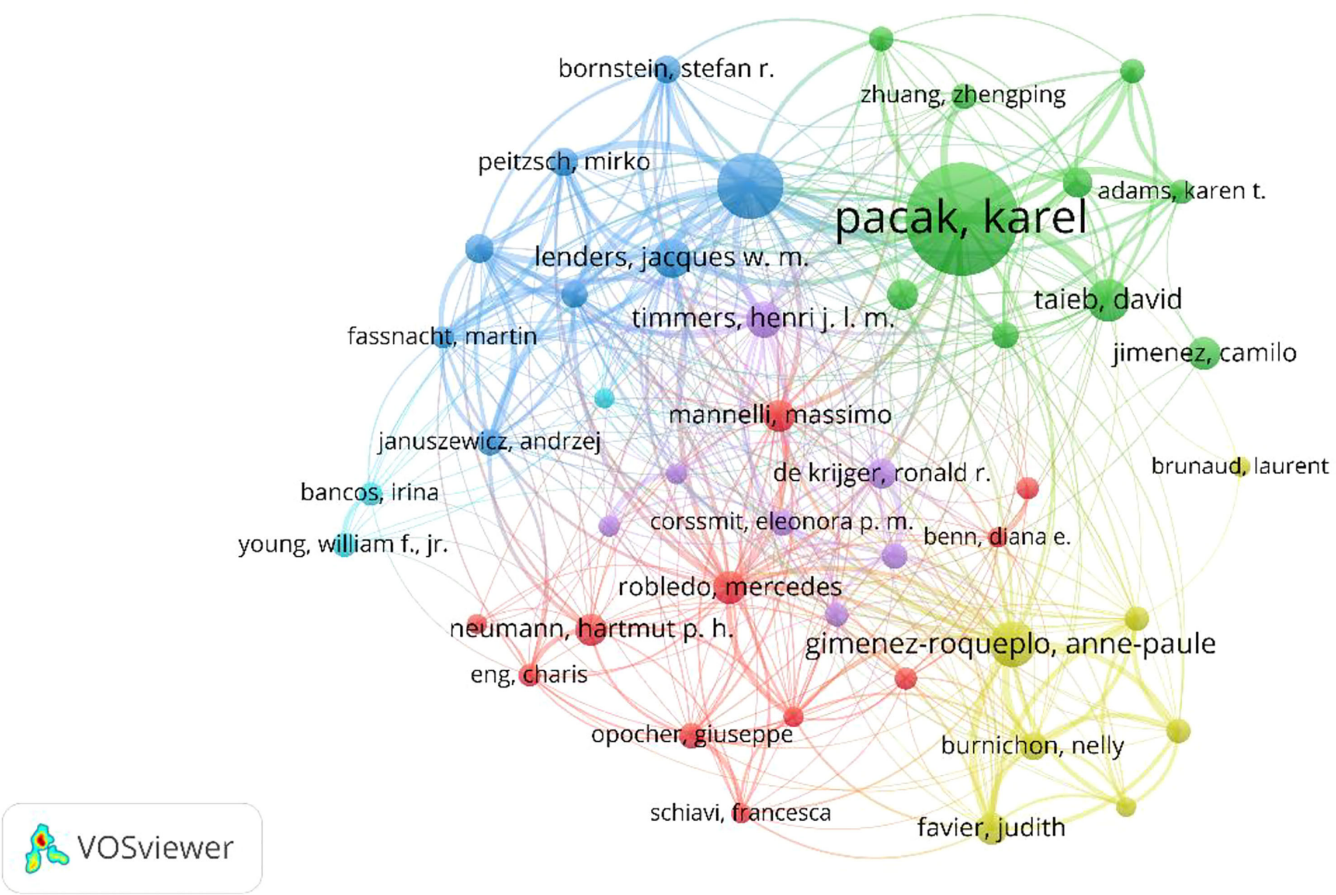
Co-authorship and network visualization of the 51 identified authors in Pheochromocytoma, with the minimum number of documents of authors was set to 20.

**Figure 7** shows the association between 901 authors (the minimum number of documents for an author is over twenty). The top 10 authors by total link strength were as follows: Pacak Karel (total link strength = 1,182), Eisenhofe Graeme (total link strength = 700), Gimenez-roqueplo Anne-paule (total link strength = 437), Timmers Henri j.l.m (total link strength = 358), Lenders Jacques w.m. (total link strength = 346), Robledo Mercedes (total link strength =346), Taieb David (total link strength = 314) and Prejbisz Aleksander (total link strength = 300), Mannelli Massimo (total link strength = 280) and Beuschlein Felix (total link strength = 262). Therefore, Pacak Karel was the most cooperative author, according to the co-authorship study.

### 3.6 Top ten most cited publications and co-cited references

**Figure 8:**
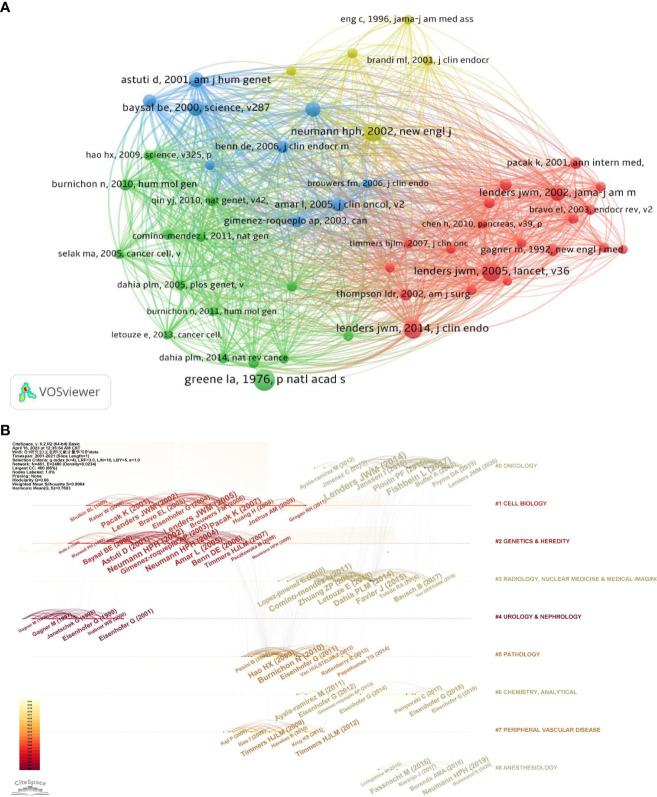
Mapping based on co-cited references from Pheochromocytoma-related research **(A)** network diagram of co-cited references, with the minimum number of citations of cited reference was set to 150 **(B)** The top nine clusters timeline distribution.

The top 10 most cited publications are listed in **Table 5**. The top ten publications were published between 2001 and 2016, which received from 645 to 1,259 citations. **Figure 8A** shows the association between 1,000 identified publications (the minimum amount of citations for a publication is over 150). According to total link strength, the top ten publications were as follows: Neumann HPH et al. (16) (total link strength = 13,904), B E Baysal et al. (17) (total link strength = 13,009), Lenders Jacques w.m. et al. (18) (total link strength = 11,798), Astuti D et al. (19) (total link strength = 11,185), Neumann HPH et al. (20) (total link strength = 11,133), Lenders Jacques w.m. et al. (1) (total link strength = 10,891), Laurence Amar et al. (21) (total link strength = 9,792), Niemann S et al. (22) (total link strength = 9,427), Lenders Jacques w.m. et al. (23) (total link strength = 9,377) and Nelly Burnichon et al. (24)(total link strength = 8,815).

**Table 5:** Top 10 most cited publications in the Pheochromocytoma research field.

Rank	Title	Year	Type	IF	Times cited
1	Lenders JW, Duh QY, Eisenhofer G, et al. Pheochromocytoma and paraganglioma: an endocrine society clinical practice guideline. J Clin Endocrinol Metab.2014;99(6):1915-1942.	2014	Article	6.134	1,259
2	Lenders JW, Eisenhofer G, Mannelli M, Pacak K. Phaeochromocytoma. Lancet. 2005;366(9486):665-675.	2005	Review	202.731	1,081
3	Neumann HP, Bausch B, McWhinney SR, et al. Germ-line mutations in nonsyndromic pheochromocytoma. N Engl J Med. 2002;346(19):1459-1466.	2002	Article	176.079	958
4	Astuti D, Latif F, Dallol A, et al. Gene mutations in the succinate dehydrogenase subunit SDHB cause susceptibility to familial pheochromocytoma and to familial paraganglioma Am J Hum Genet. 2001;69(1):49-54.	2001	Article	11.043	816
5	Lenders JW, Pacak K, Walther MM, et al. Biochemical diagnosis of pheochromocytoma: which test is best?. JAMA. 2002;287(11):1427-1434.	2002	Article	157.335	751
6	Fassnacht M, Arlt W, Bancos I, et al. Management of adrenal incidentalomas: European Society of Endocrinology Clinical Practice Guideline in collaboration with the European Network for the Study of Adrenal Tumors. Eur J Endocrinol. 2016;175(2): G1-G34.	2016	Article	6.558	721
7	Isaacs JS, Jung YJ, Mole DR, et al. HIF overexpression correlates with biallelic loss of fumarate hydratase in renal cancer: novel role of fumarate in regulation of HIF stability. Cancer Cell. 2005;8(2):143-153.	2005	Article	38.585	681
8	Young WF Jr. Clinical practice. The incidentally discovered adrenal mass. N Engl J Med. 2007;356(6):601-610.	2007	Article	176.079	672
9	Neumann HP, Pawlu C, Peczkowska M, et al. Distinct clinical features of paraganglioma syndromes associated with SDHB and SDHD gene mutations [published correction appears in JAMA. 2004 Oct 13;292(14):1686]. JAMA. 2004;292(8):943-951.	2004	Article	157.335	655
10	Pollard PJ, Brière JJ, Alam NA, et al. Accumulation of Krebs cycle intermediates and over-expression of HIF1alpha in tumours which result from germline FH and SDH mutations. Hum Mol Genet. 2005;14(15):2231-2239.	2005	Article	5.121	645

The reference timeline view can be used to illustrate how research hotspots have changed over time. The terms used the most frequently in each cluster were chosen as the label for that cluster. As seen in **Figure 8B**, clusters #1 (cell biology), clusters #2 (genetic and heredity) and #4 (urology and nephrology) began earlier, while clusters #0 (oncology), #3 (radiology, nuclear medicine and medical imaging), #5 (physiology), #6 (chemistry, analytical), and #7 (peripheral vascular disease) and #8 (anesthesiology), were still being studied in recent years.

**Figure 9:**
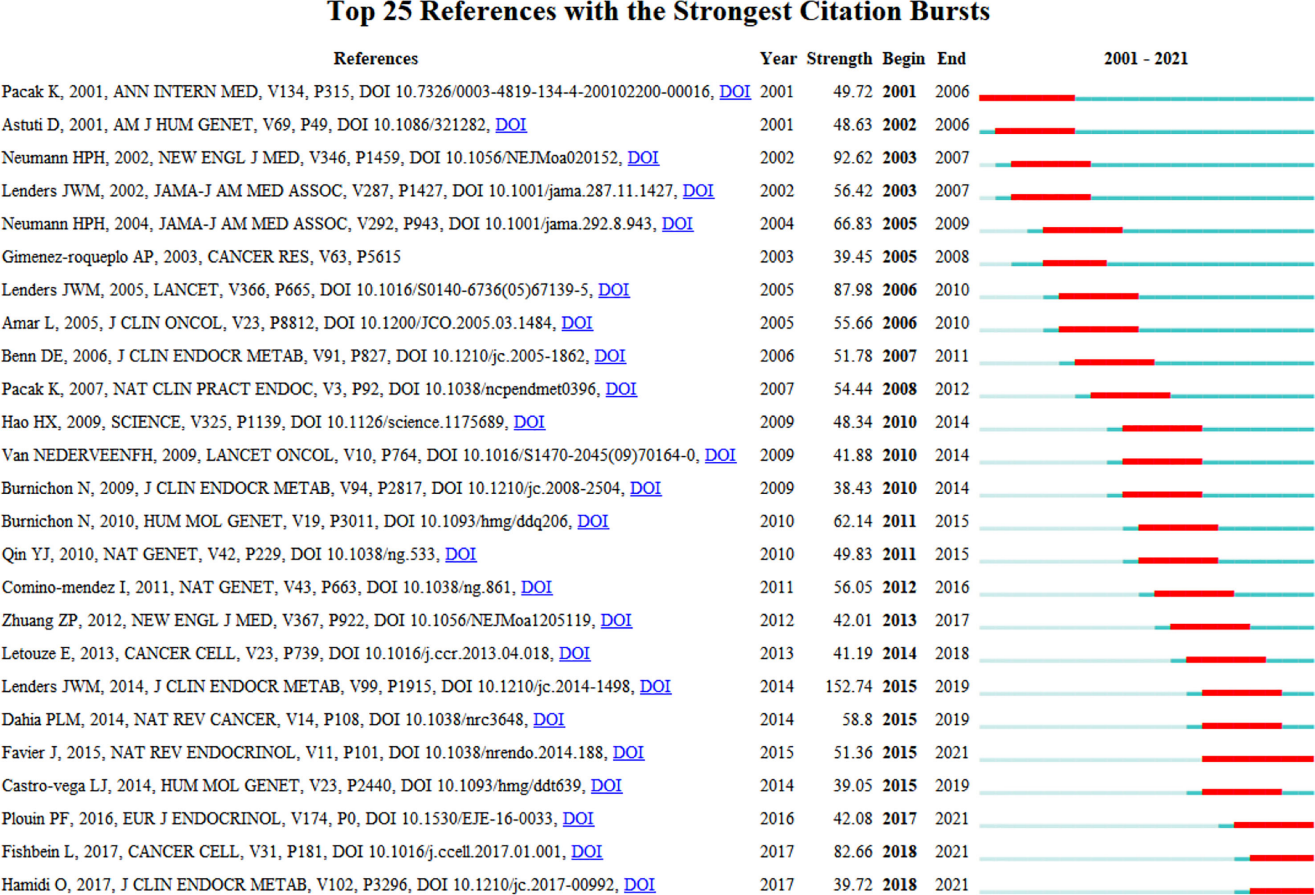
The top 25 co-cited references with the most citation burstiness. The years between “Begin” and “End” represent the period when the reference was more influential. Years in light green mean that the reference has not yet appeared, years in dark green mean that the reference is less influential, and years in red mean that the reference is more influential.

The top 25 references with the most powerful citation bursts were shown in **Figure 9**. Lenders, JWM.’s (18) and Neumann, HPH’s publications (16) have the highest strength. It is consistent with the results of the top ten references analysis in the previous section. Furthermore, in recent years, the citation times of articles by Fishbein L (25) and Hamidi (26) have greatly increased. It demonstrated that their research had captured the interest of other researches worldwide recently.

### 3.7 Analysis of hot spots in research

**Figure 10:**
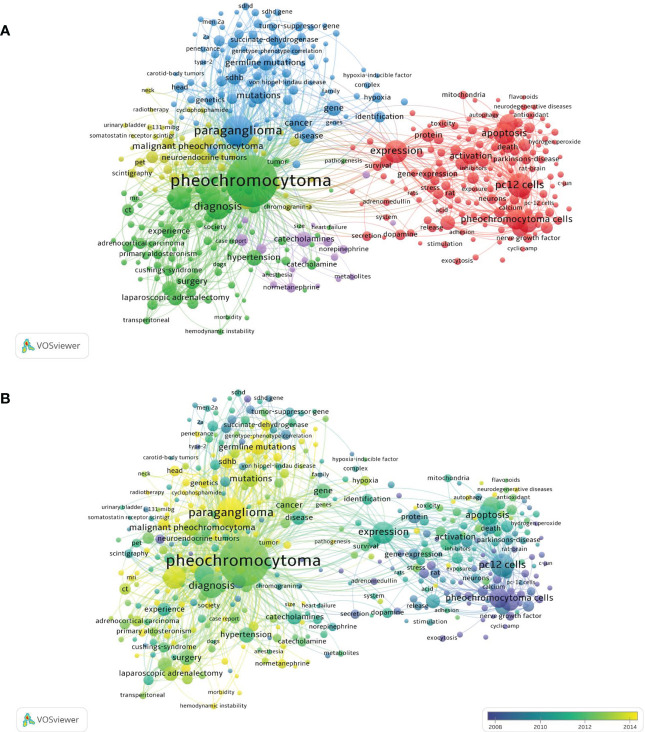
Keyword maps globally **(A)** Keyword maps in the past 20 years with the number of occurrence of keywords was set to 30 **(B)** Distribution of keywords according to their time of appearance. Keywords in blue and purple appeared earlier than those in yellow.

**Figure 11:**
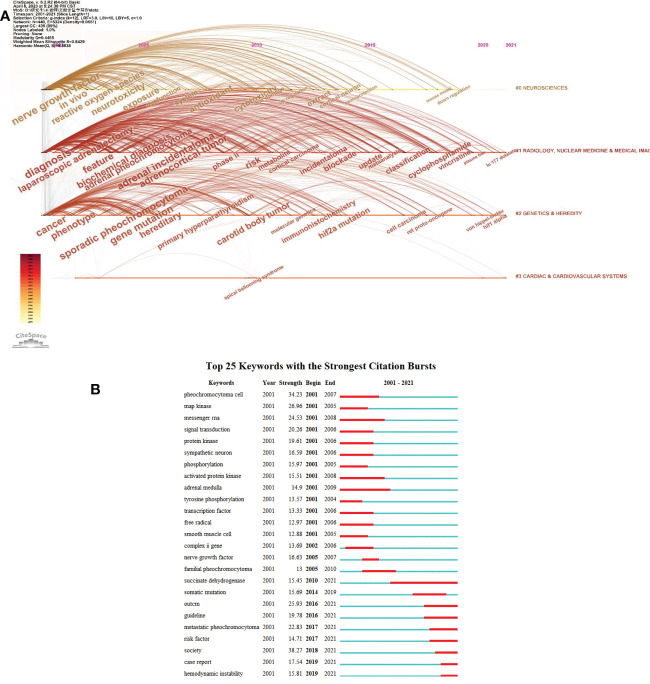
Popular keyword analysis **(A)** Timeline distribution of keyword cluster analysis **(B)** The top 25 Mesh terms and their outbreak time (Strength reflects the frequency of occurrence of the keyword. The years between “Begin” and “End” represent the period when the keyword was more influential. Years in red mean that the keyword is more influential.

To identify the hot research directions and subjects important for following the development of science, VOSviewer and Citespace were used to assess keywords gathered from the titles and abstracts of 8653 articles. (**Figures 10**, **11**). As shown in **Figure 10A**, the 1000 detected keywords are grouped into five clusters: green clusters represent “Treatment study”, mainly about surgeries; blue clusters represent “Etiology study”, mainly about genetics and heredity; red clusters represent “Mechanism study”, mainly about neurosciences; yellow clusters represent studies on “Radiological study”, mainly about radiology, nuclear medicine and medical imaging; and purple clusters represent “Hormones study”. Distribution of keywords according to their time of appearance were shown in **Figure 10B**, keywords are colored according to the average time they appear in publications on average. Keywords in blue/purple appeared earlier than those in yellow.

**Figure 11A** shows the keyword clusters, the interrelationship between the clusters and the change of each keyword over time. **Figure 11B** shows the top 25 items’ concentration period and how hot spots have changed over time from 2001 to 2021. The words “succinate dehydrogenase,” “metastatic pheochromocytoma,” and “hemodynamic instability,” were found to be the most recently trending terms.

## 4 Discussion

A total of 8653 pheochromocytoma-related literature, including 7,607 articles and 1,046 reviews published from 2001 to 2021, were included in our study. The number of publications on pheochromocytoma has been relatively stable over the last 20 years. The number of publications in China is gradually increasing, while the number of publications in Japan is steadily decreasing. In contrast, the number of publications in other countries has remained stable (**Figure 2**).

During the past 20 years, the quantity of publications on pheochromocytoma has been largely steady. It is anticipated that the pheochromocytoma-related publications will remain steady in the coming years. The national distribution made it clear that pheochromocytoma research was conducted by scientists all over the world, especially in the USA. Chinese contribution was less than that of the USA before 2020 and gradually overtook USA after 2020. It may be attributed to the rapid development of China and the increasing annual funding for pheochromocytoma research.

USA, with the highest H-index, published the most among countries with 2,777 documents and 39.24 average citations, followed by China, Japan, Germany, Italy, France, England, Netherlands, South Korea and Canada (**Table 1**). What’ more, co-authorship analysis (**Figure 3**) revealed that authors from USA were more cooperative than authors from other countries. Therefore, our results demonstrated that the USA was the top country in Pheochromocytoma research globally and was more willing to cooperate with other countries.

From the perspective of institutions, Eunice Kennedy Shriver Natl Inst Child Hlth & Hum published the most of the articles (**Table 2**) and has the strongest relationships with other institutions (**Figure 4**), showing that this association has high academic credentials and is more eager to work with others while National Institute of Child Health and Human Development ranks first in terms of the average citation numbers. The majority of these highly productive institutions in the field of pheochromocytoma are from the United States, and these findings further illustrate the top position of the United States in the contribution to the field of pheochromocytoma.

The most cited journals and one that published the majority of the pheochromocytoma research was *Journal of Clinical Endocrinology & Metabolism*, indicating that it was the leading journal at Pheochromocytoma worldwide (**Table 3**). In addition, co-citation analysis (**Figure 5**) also showed that the *Journal of Clinical Endocrinology and Metabolism* was the most popular journal for pheochromocytoma-related research worldwide. The *Journal of Neurochemistry* and the *Journal of Biological Chemistry* both ranked in the top 5 for publications and average citations, highlighting their significant contributions to the advancement of pheochromocytoma research.

Furthermore, the primary citation fields for pheochromocytoma are molecular, biological, and immunological study (**Figure 6**). In addition, these journals were ranked in the top 10 for the following possible reasons: first, the scientific directions and research topics they cover are most pertinent to the topic of pheochromocytoma, so scientists are easily encouraged to submit relevant research to these journals. Second, these journals are the most prominent professional journals assisting researchers in enhancing their scientific ideals and perspectives and improve their academic standards and research capabilities through discussions and exchanges with their peers. In addition, these journals are more well-liked by researchers because the relatively short peer review cycles.

As shown in **Table 4**, the three authors who had the most articles published on pheochromocytoma research and had highest H-index were Pacak, karel, Eisenhofer, graeme, and Gimenez-roqueplo, anne-paule. In terms of average citation, Gimenez-roqueplo and anne-paule have the highest average citation. Thus, Pacak Karel, Eisenhofer, graeme, and Gimenez-roqueplo, anne-paule were the most productive author, while Gimenez-roqueplo, anne-paule published the highest quality articles on average in pheochromocytoma. These highly productive authors come from the United States, Germany, France, the Netherlands, Spain, and Italy. The countries to which these high-producing authors belong are almost identical to the countries of the high-producing institutions described above. Therefore, more consideration and importance should be given to these authors in order to keep up with innovations in this field. As for co-authorship analysis shown in **Figure 7**, Pacak Karel has the largest total link strength, indicating that Pacak Karel is not only the productive but also the most cooperative author.

According to the top ten most cited articles shown in **Table 5**, Lenders, JWM. et al. Wrote the first, second and fifth most cited publications, which comprehensively summarized the clinical presentation, diagnosis and treatment of pheochromocytoma (1, 18, 23). In addition, Neumann, HPH et al. published the third and tenth most cited articles, both of which converged on etiologic studies, describing the germline and gene mutations of pheochromocytoma (16, 20). The other top ten most cited publications included articles related to pheochromocytoma on genetic mutations and treatments, either. Additionally, most of these publications were published before 2010. It suggested that the recent publications take time to receive widespread citations. What’s more, Neumann HPH et al. have the strongest correlations with others according to the co-citation analysis (**Figure 8A**), which indicates that the article published by Neumann HPH et al. (16). has the greatest global impact.

Timeline visualization of the references and keywords (**Figures 8B**) showed that multidisciplinary sciences, cell biology and medical laboratory technology were still being studied in recent years. Among them, in cell biology, an article published by Dahia PLM in 2020 mentions the important role of hypoxia in the pathogenesis of pheochromocytoma (27). In medical laboratory technology, an article by Eisenhofer G published in 2021 demonstrates improvements in pheochromocytoma biomarker detection method (28). As for the analysis of references with the strongest citation bursts (**Figure 9**), in recent years, the citation times of articles by Fishbein L (25). and Hamidi (29) have greatly increased. Their articles illustrate the importance of individualized treatment of pheochromocytoma in terms of Molecular Characterization and clinical presentation, respectively. This indicates that precision medicine research on pheochromocytoma will be a hot spot for future research.

By analyzing the number of articles whose keywords occurred together in titles or abstracts, a co-occurrence network visualization is produced. The goal is to identify the hot research directions and subjects important for following the development of science (**Figures 10**, **11**). As shown in **Figure 10A**, the most frequently used keywords in the “Treatment study” cluster were pheochromocytoma, surgery, adrenalectomy and laparoscopic adrenalectomy. The most commonly used terms for the “Etiology study” cluster were paraganglioma, germline mutation, genetics and succinate dehydrogenase subunit B(SDHB). whereas the pheochromocytoma cells, apoptosis and nerve growth-factor were the most often used keywords in the “Mechanism study” cluster. The primary keywords for the “Radiological study” cluster were positron-emission-tomography, scintigraphy, and radiotherapy. The primary keywords in the cluster “Hormones study” were catecholamines, normetanephrine, norepinephrine, and metabolites.

These keywords have significant importance in their respective fields and had a greater overall link strength. These findings might offer fresh perspectives on pheochromocytoma’s popular research directions and themes, showing which areas warrant more investigation and high-caliber research in the future.

In **Figure 10B**, keywords are colored according to the average time they appear in publications on average. Keywords in blue appeared earlier than those in yellow. Co-occurrence analysis results showed that “Radiology study” and “Etiology study” may become the focus of future pheochromocytoma research. In addition, the overlay visualization included a number of cutting-edge terms relevant to the pathogenesis of pheochromocytoma, such as germlines mutation, mesenchymal stem-cells, autophagy, neuroinflammation, neurotoxicity, and hemodynamic instability. Recent research found that Exosomes from adipose mesenchymal stem cells improve pheochromocytoma cell (PC12 cell) function by activating the phosphatidylinositol 3-kinase (PI3K)/protein kinase B (PKB/AKT) pathway, which was a key pathophysiology of pheochromocytoma (30). What’ s more, Hye Min Kim et al. Showed that autophagy-related proteins are differentially expressed in pheochromocytoma patients and correlate with patient prognosis (31). Additionally, the role of neuroinflammation and neurotoxicity in the pathogenesis of pheochromocytoma has been gradually increasing in recent years (32, 33). Therefore, these directions deserve more time and funding in the future for more in-depth and comprehensive research.

Keyword clusters and the interrelationship between the clusters and the change of each keyword over time were shown in **Figures 11A**. It showed that neurosciences, genetics& heredity, and radiology, nuclear medicine & medical imaging were still being studied in recent years. **Figure 11B** shows the top 25 items’ concentration period and how hot spots have changed over time from 2001 to 2021. The words “succinate dehydrogenase,” “metastatic pheochromocytoma,” and “hemodynamic instability,” were found to be the most recently trending terms. In another word, the researchers have paid great attention to succinate dehydrogenase, metastatic pheochromocytoma and hemodynamic instability related to pheochromocytoma recently.

Our study is the first bibliometric study to analyze the pheochromocytoma-related research, which provides an overview for researchers to better understand the hotspot, status and trends in pheochromocytoma-related research. However, the limitations should also be acknowledged: First, we only used the WOS database for literature data search, which may have an impact on the accuracy of the study results. Second, we only examined English-language publications, which may also lead to a language bias. Third, only the literature from 2001 to 2021 was included due to limitations in the volume of literature.

## 5 Conclusion

Our study identifies pheochromocytoma-related publications over the past 20 years and introduces their global trends and status. Although the number of articles on pheochromocytoma has fluctuated slightly over the past 20 years, there has been an overall upward trend. USA was the leading country of pheochromocytoma in terms of total publications and average citations. The leading institutions that contributed to pheochromocytoma were Eunice Kennedy Shriver Natl Inst Child Hlth & Hum, National Cancer Institute and Mayo Clinic. The main publications for pheochromocytoma-related articles were *Journal of clinical endocrinology &metabolism, Journal of neurochemistry* and *Endocrine-related cancer*. Moreover, Pacak karel, Eisenhofer Graeme and Gimenez-roqueplo Anne-paule were the main contributing authors to the field. In general, precision medicine research on pheochromocytoma, especially metastatic pheochromocytoma, in terms of diagnosis, treatment, and etiology will be a hot research topic in the future. Specifically, germlines mutation, mesenchymal stem-cells, autophagy, neuroinflammation, neurotoxicity, and hemodynamic instability are the keywords that have attracted more attention from researchers in recent years.

## Data availability statement

The original contributions presented in the study are included in the article/supplementary material. Further inquiries can be directed to the corresponding authors.

## Author contributions

MW and YZ decided and conceptualized this article, and revised the draft. B-LH collected, analyzed the data and wrote the manuscript. QL, Y-yT, S-qP and ZL collected and analyzed the data. M-lL and J-yL prepared the figures and tables. YZ and MW was the guarantor of the overall content. All authors approved the final version of the manuscript and agreed to be accountable for all specs of the work. All authors contributed to the article and approved the submitted version.
